# Evaluation of pregnancy outcomes using medroxyprogesterone acetate versus gonadotropin-releasing hormone antagonist in ovarian stimulation: A retrospective cohort study

**DOI:** 10.18502/ijrm.v20i6.11445

**Published:** 2022-07-06

**Authors:** Ekika Singh, Christophe Blockeel, Madhulika Singh, Rishi Gupta, Sandesh Kamdi

**Affiliations:** ^1^Sharda Narayan Hospital, Mau, UP, India.; ^2^Centre for Reproductive Medicine, Laarbeeklaan, Brussels, Belgium.; ^3^Manokalp Clinic, Delhi, India.; ^4^Pacific Academy of Higher Education and Research University, Udaipur, Rajasthan, India.

**Keywords:** Oocyte donation, Medroxyprogesterone acetate, Gonadotropin-releasing hormone antagonist, Pregnancy outcomes.

## Abstract

**Background:**

Limited studies have compared pregnancy outcomes with medroxyprogesterone acetate (MPA) vs. gonadotropin-releasing hormone antagonist (GnRH antagonist) in ovarian stimulation protocols. The results show heterogeneity.

**Objective:**

This study aims to assess pregnancy outcomes with the use of MPA instead of GnRH antagonist for ovarian stimulation in donor-recipient cycles.

**Materials and Methods:**

This retrospective study was carried out from June 2016 to May 2019. The study included 250 donors receiving ovarian stimulation with 2 different protocols: group 1 (n = 109) receiving GnRH antagonist (0.25 mg/day) from the 5 ${}^{\text{th}}$ or 6 ${}^{\text{th}}$ day of menses and group 2 (n = 141) receiving MPA (10 mg/day) from the second day of menses. In 384 recipients, 2 good-quality blastocysts were transferred after endometrial preparation. The primary endpoint was live birth in recipients.

**Results:**

The results showed that live birth was comparable in both recipient groups (59% vs. 60%, OR: 0.63, 95% CI: 0.13-2.99, p = 0.559). The number of live-born fetuses (adjusted OR: 0.57, 95% CI: 0.31-1.05, p > 0.01) showed no significant difference in both groups. However, the implantation rate with twin sacs was significantly lower in group 2 (adjusted OR: 0.57, 95% CI: 0.33-0.99, p = 0.05). The regression analysis for good-quality blastocyst proportion was comparable (OR: 0.63, 95% CI: -4.33-5.60, p = 0.802) in both donor groups. The mean stimulation cost in group 2 was less than in group 1.

**Conclusion:**

MPA had a comparable live birth and embryological outcomes in both groups. Oral administration makes it convenient, acceptable, and patient-friendly. Its cost-effectiveness and convenience open new possibilities in ovarian stimulation protocols.

## 1. Introduction

Luteinizing hormone (LH) surges and, hence, ovulation can be blocked by progesterone. It has been shown that giving progestin from the start of the cycle blocks LH surge despite a rise in estradiol, as long as it is given. It sharply decreases both follicle-stimulating hormone (FSH) and LH secretion. This inhibition is completely reversible after discontinuation of progestin (1, 2). It has been shown that progestin blocks LH surges due to consistent LH suppression during ovarian stimulation (OS) (3-5). Medroxyprogesterone acetate (MPA) is an effective oral alternative for gonadotropin-releasing hormone (GnRH) analogue injections to prevent LH surges. Comparative studies of progestin for the prevention of LH surge, and its subsequent embryological and pregnancy outcomes in patients undergoing OS, followed by fresh embryo transfer (FET) in the next cycle have shown similar results and comparable outcomes (2, 5). Limited studies have compared GnRH antagonist vs. MPA in oocyte donation cycles. Although steroidal preparations are novel in their application for a successful in vitro fertilization (IVF) treatment they have a negative impact on the endometrium and its receptivity and need cryopreservation of embryos (6, 7). However, in the case of oocyte donation cycles, endometrial receptivity is not necessary and embryos can be transferred to the recipients in the same cycle after adequate preparation of the endometrium. Thus, this study compares the effect of GnRH antagonist vs. MPA on various reproductive parameters for successful pregnancy outcomes. The present work was carried out to compare the results of pregnancy outcomes in recipients and present current possibilities of using gestagens instead of GnRH antagonists in OS protocols.

## 2. Materials and Methods

### Study design 

This retrospective cohort study was carried out from June 2016 to May 2019 at a tertiary level hospital located in Mau, Uttar Pradesh, India. Overall, 250 oocyte donors meeting the requirements for a donation of gametes as per Indian Council of Medical Research/National Academy of Medical Sciences (India) guidelines (ICMR/NAMS 66.3.7) were included. Egg donors were between 21 and 28 yr old and free of HIV and hepatitis B and C infections, hypertension, diabetes, sexually transmitted diseases, or any other identifiable and common genetic disorders such as thalassemia. Their blood group, the Rhesus status, height, weight, body mass index (BMI), educational qualifications, profession, the color of the skin and the eyes, and the family background concerning the history of any familial disorder were recorded. All the donors were randomly allocated to the groups. While in group 1, 109 donors received GnRH antagonists, 141 in group 2 received MPA. The inclusion criteria for recipient females were menopause, primary ovarian insufficiency irrespective of age, poor response in 2 previous cycles, and recurrent implantation failure. The exclusion criteria for recipients included normal and hyper responder, uncontrolled endocrine, or any other medical disorder.

### Protocol for OS (oocyte donors)

In group 1, OS was started with recombinant follicle-stimulating hormone (rFSH, Folisurge ${}^{\text{TM}}$ , Intas Pharmaceuticals Ltd., India) at 225-300 IU/day and GnRH antagonist (Asporelix ${}^{\text{TM}}$ , 0.25 mg injection - Bharat Serums & Vaccines Ltd., India) using the flexible protocol, initiated on day 5 or day 6 when the leading follicles reached > 13 mm and continued until the trigger day. In group 2, stimulation was performed by combining rFSH at 225-300 IU/day with 10 mg/day MPA (Maxogest ${}^{\text{TM}}$ tablet, Corona Remedies Pvt. Ltd., India) at the same time as rFSH was initiated. In both groups, Triptorelin 0.2 mg S.C. (Decapeptyl ${}^{\text{TM}}$ , 0.1 mg/day, Ferring Pharmaceuticals, India) was given as soon as a major cohort of follicles > 18 mm was observed. Transvaginal ultrasound-guided oocyte retrieval was carried out 35-36 hr after the trigger. Oocytes were denuded of cumulus cells and M2 were allocated to recipients in both groups. Oocytes were inseminated by intracytoplasmic sperm injection (ICSI) after selecting sperms. Embryos were cultured in 1-step media until the blastocyst stage. Gardner's grading system was used for grading blastocysts. Embryos of the 3AA stage and above were labeled as good-quality blastocysts. FET of 2 good-quality blastocysts was performed in recipients. The recipient's endometrium was prepared using an artificial hormonal replacement cycle.

### Hormone replacement therapy (recipients)

Endometrial preparation was started in all recipient patients from day 2 of menses. 6 mg/d of estradiol valerate (Progynova ${}^{\text{TM}}$ , 2 mg tablet, Zydus Cadila, Pharmaceuticals, India) was started on the second day of the menstrual cycle to achieve an endometrial thickness of > 7.0 mm. In cases where inadequate endometrial thickness was observed, the dose of estradiol valerate was increased up to 12 mg to achieve > 7 mm endometrial thickness. On the oocyte retrieval day of the donor, a progesterone injection of 100 mg/ml/day (Gestone ${}^{\text{TM}}$ injection, Ferring Pharmaceuticals, India) was administered to the recipient along with estradiol valerate. Day 5 embryo transfer was performed with fresh embryos. 2 good-quality blastocysts were transferred. Estradiol valerate and progesterone were continued at the same dose for 2 wk following the transfer. Serum β human chorionic gonadotropin was performed 2 wk later; in the case of positive values, the same treatment was continued until 12 wk of gestation.

### Outcome measures

The primary outcome was the live birth rate in recipients. Secondary outcomes related to donors included M2 oocyte proportion percent at oocyte retrieval, duration of stimulation, total consumption of gonadotropins, blastulation rate, and good-quality blastocyst proportion. Secondary outcomes related to recipients were biochemical pregnancy, implantation rate, and clinical pregnancy rate.

### Ethical considerations

The study protocols were approved by Sharda Narayan Ethics Committee, Mau, India (SNH/Ethics/001), and informed written consent was taken from each participant.

### Statistical analysis

Microsoft Excel was used to code and record data. SPSS v23 (IBM Corp., released 2015, IBM SPSS Statistics for Windows Version 23.0, Armonk, NY: IBM Corp.) was used for data analysis. Means / standard deviations and medians / interquartile ranges were calculated for continuous variables, forms were used for expressing descriptive analyses and categorical variables were expressed as frequencies and percentages. The independent sample *t* test was used for group comparison of continuously distributed data when comparing 2 groups. In the case of uneven distribution, appropriate non-parametric tests, including Wilcoxon test and Kruskal Wallis test, were used for comparisons. Categorical data were analyzed using the Chi-square test for group comparisons. Binary logistic regression was used to ascertain the significant predictors of dichotomous outcomes and linear regression for continuous ones. The individual coefficients/odds ratios and significance for each of the variables were first performed using univariable regression, then backward stepwise variable selection was used to choose the best set of predictor variables. Finally, multivariable linear/logistic regression was performed to get the final model. Statistical significance was kept at p < 0.05.

## 3. Results

A total of 250 oocyte donors fulfilling all the criteria of oocyte donation as per Indian Council of Medical Research guidelines, who received an OS with 2 protocols, were studied. The pregnancy outcomes in 384 recipients who received oocyte donation or embryo donation (using donor sperm and donor oocyte) from donors using 2 different OS protocols were studied. Cycle characteristics of the recipient groups are depicted in table I. The mean age of recipients was 38.80 ± 6.37 and 38.39 ± 6.32 yr in both groups, respectively; BMI and Anti-Mullerian hormone were comparable in both groups (p = 0.47, p = 0.17, respectively). Sperm source (self or donor) was statistically significant (p = 0.019) with more self-sperm ICSI in group 2. Indication for IVF had a significant difference (p = 0.023) in the two groups. All oocytes retrieved from both donor groups were inseminated by ICSI. Out of the 181 recipients in group 1, donor sperm was used in 77 recipients, while out of the 203 recipients in group 2, donor sperm was used in 66 recipients. 2 good-quality blastocysts were transferred on day 6 of progesterone supplementation in both groups. On analysis, as depicted in table II, biochemical pregnancy was 74.0% vs. 73.4% in groups 1 and 2, respectively. Implantation and clinical pregnancy were comparable in both groups. The implantation rate with twin sacs was significantly lower in group 2. Out of the 110 ongoing pregnancies, 107 were delivered in group 1. There were 123 deliveries from 130 ongoing pregnancies in group 2. Table III depicts the regression models for all pregnancy outcomes in both the univariable and multivariable analyses. Univariable and multivariable regression was done using group 1, group 2, sperm source, age, BMI, recurrent implantation failure, and endometrial thickness as predictors and pregnancy outcomes as a dependent variable. The group 1 categorical variable was used as the reference category.

On comparing group 1 (GnRH antagonist) with group 2, the univariable analysis provided an unadjusted OR of 0.97 (95% CI: 0.61-1.53, p = 0.893) for biochemical pregnancy, 1.02 (95% CI: 0.42-2.49, p = 0.962) for implantation, 1.78 (95% CI: 0.57-5.61, p = 0.322) for clinical pregnancy, 2.67 (95% CI: 0.80-8.90, p = 0.111) for ongoing pregnancy, and 0.49 (95% CI: 0.12-1.96, p = 0.316) for live birth. The multivariable regression analysis of the above dependent variables was not statistically different. The unadjusted OR of 0.57 (95% CI: 0.33-1.00, p = 0.049) for the implantation rate of twin sacs was significantly less in group 2 (MPA). The multivariable adjusted OR for the implantation rate provided a statistically significant difference in twin sac implantation. However, live birth and the number of live-born fetuses were not significantly different in both groups. The multivariable adjusted OR of 0.57 (95% CI: 0.31-1.05, p = 0.073) for live-born fetuses showed no significant difference as depicted in table IV. Demographic details and cycle characteristics of the donor groups are in table V. The mean age and BMI of donors showed no significant difference in both groups. The duration of stimulation was 11.03 ± 0.80 days in group 1 and 11.0 ± 0.74 days in group 2. Gonadotropin consumption in group 1 was 2428 ± 278 and in group 2 was 2251 ± 141 (p = 0.400). The M2 oocytes retrieved in both groups were 14.60 ± 8.26 and 13.40 ± 6.53, respectively. The blastulation rate was better in group 2 but this was not statistically significant (p = 0.165). All day 5 embryos reaching the morphological stage of 3AA and above (according to the Gardner staging system) were labeled as good-quality blastocysts. Good-quality blastocysts were comparable in both groups. Regression analysis was performed for M2 oocyte proportion percent and good-quality embryo proportion percent as the dependent variable. Group 1, group 2, age, BMI, stimulation dose, and retrieved oocytes were taken as predictors (Table VI). M2 oocyte proportion percent univariable analysis (OR: 0.79, 95% CI: -1.74 to 3.32, p = 0.538) did not show a significant difference in the groups. On further multivariable analysis, though there was a slightly higher M2 oocyte proportion percent in group 2, the difference was not statistically significant (OR: 1.08, 95% CI: -1.47 to 3.62, p = 0.406). The retrieved oocyte number independently affected the M2 oocyte proportion percent, after adjusting for confounding factors in both univariable analyses (OR: 0.01, 95% CI: 0.00-0.01, p = 0.002) and multivariable analyses (OR: 0.01, 95% CI: 0.00-0.01, p = 0.005); however, the regression analysis for good-quality blastocyst proportion was comparable using both univariable and multivariable analysis in between the groups. It can be concluded from the results of regression analysis that embryological outcomes especially M2 oocyte proportion and good-quality blastocysts proportion were comparable in both the groups.

**Figure 1:**
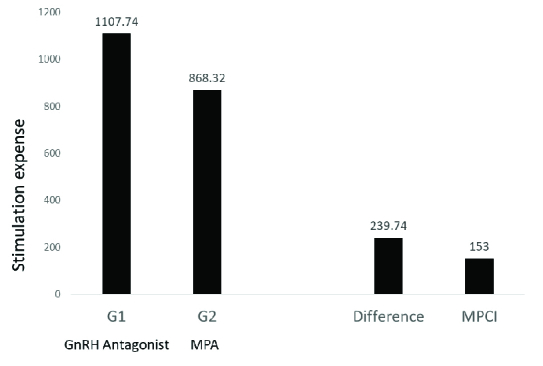
Stimulation cost in both groups, MPCI: Monthly per capita income, MPA: Medroxyprogesterone acetate, GnRH: gonadotropin-releasing hormone.

The mean stimulation cost for the rFSH and GnRH antagonist protocol in group 1 was $1,107.74 while in group 2 with rFSH and MPA protocol, it was $868.82. A difference of $239.74 was observed in stimulation costs, with the MPA protocol being more cost-effective. With a monthly per capita income of $153 in India, OS with MPA appears to be a more effective and convenient option as shown in figure 1.

**Table 1:** Demographic and cycle characteristics of recipients groups


**All variables**		**Groups**		**P-value**
		**Group 1 (n = 181)**	**Group 2 (n = 203)**	
**Sperm source ${}^{\text{a}}$ **				
	**Self**	104 (57.5)	140 (69.0)	
	**Donor**	77 (42.5)	63 (31.0)	0.019 ${}^{*}$
**Age (yr) ${}^{\text{b}}$ **		38.82 ± 6.37	38.39 ± 6.31		0.474 ${}^{**}$
**BMI ${}^{\text{b}}$ (kg/m^2^)**		25.62 ± 3.66	25.46 ± 4.24	0.171 ${}^{**}$
**AMH ${}^{\text{b}\#}$ (ng/ml)**		1.04 ± 2.05	1.01 ± 1.32	0.527 ${}^{**}$
**Indication ${}^{\text{a}}$ **				
	**PM**	84 (46.41)	83 (40.89)	
	**POR**	58 (32.04)	50 (24.63)	
	**POI**	25 (13.81)	37 (18.23)	
	**RIF**	14 (7.73)	33 (16.26)	0.023 ${}^{*}$
**Endometrial thickness (mm)**		8.53 ± 1.10	9.41 ± 8.03		0.180 ${}^{**}$
${}^{\text{a}}$ Data shown as n (%), ${}^{\text{b}}$ Data shown as Mean ± SD. *Chi-square test, Significant at p < 0.05, **Wilcoxon Mann-Whitney U test, BMI: Body mass index, AMH: Anti-Mullerian hormone, PM: Post-menopausal, POR: Poor ovarian reserve, POI: Primary ovarian insufficiency, RIF: Recurrent implantation failure, ${}^{\#}$ OR (IQR): Group 1: 0.87 (0.5-1.1), Group 2: 0.79 (0.52-1.1)				

**Table 2:** Pregnancy outcomes of 2 the recipient groups


**All variables**		**Group**		**P-value**
		**1 (n = 181)**	**2 (n = 203)**	
** β HCG**				
	**Negative**	47 (26.0)	54 (26.6)	
	**Positive**	134 (74.0)	149 (73.4)	0.888 ${}^{*}$
**Implantation**				
	**Absent**	10 (7.5)	11 (7.4)	
	**Present**	123 (92.5)	138 (92.6)	0.965 ${}^{*}$
Implantation rate***				
	**1 sac**	82 (66.7)	108 (78.3)	
	**2 sac**	41 (33.3)	30 (21.7)	0.036 ${}^{*}$
**Clinical pregnancy**				
	**Absent**	8 (6.5)	5 (3.8)	
	**Present**	115 (93.5)	128 (96.2)	0.318 ${}^{*}$
**Ongoing pregnancy**				
	**Absent**	9 (7.6)	4 (3.0)	
	**Present**	110 (92.4)	130 (97.0)	0.100 ${}^{*}$
**Live birth**				
	**Absent**	3 (2.7)	7 (5.4)	
	**Present**	107 (97.3)	123 (94.6)	0.351 ${}^{**}$
**Number of live fetuses**				
	**Single**	72 (67.3)	96 (78.0)	
	**Twins**	35 (32.7)	27 (22.0)	0.067 ${}^{*}$
***Significant at p < 0.05, ${}^{*}$ Chi-square test, ${}^{**}$ Fisher's exact test, βHCG: Beta human chorionic gonadotropin				

**Table 3:** Regression analysis of pregnancy outcomes


**Pregnancy outcomes**	**OR (95% CI) univariable**	**P-value**	**OR (95% CI) multivariable**	**P-value**
**Biochemical pregnancy**	0.97 (0.61-1.53)	0.893	1.05 (0.65-1.68)	0.850
**Implantation **	1.02 (0.42-2.49)	0.962	1.25 (0.49-3.21)	0.642
**Implantation rate**	0.57 (0.33-1.00)	0.049	0.57 (0.33-0.99)	0.046
**Clinical pregnancy**	1.78 (0.57-5.61)	0.322	1.70 (0.50-5.74)	0.395
**Ongoing pregnancy**	2.67 (0.80-8.90)	0.111	2.73 (0.79-9.45)	0.112
**Live birth**	0.49 (0.12-1.96)	0.316	0.63 (0.13-2.99)	0.559
**Number of live born fetuses**	0.60 (0.33-1.08)	0.090	0.57 (0.31-1.05)	0.073
Data presented as OR (95% CI). OR: Odds ratio, CI: Confidence interval, Significant at p < 0.05				

**Table 4:** Regression with all variables in the model


**Dependent: number of live-born fetuses**		**Single**	**Twins**	**OR (95% CI) univariable**	**OR (95% CI) multivariable**
**Group 1 ${}^{\text{a}}$ **		71 (67.6)	34 (32.4)	- -
**Group 2 ${}^{\text{a}}$ **		98 (79.6)	25 (20.3)	0.60 (0.33-1.08, p = 0.090)	0.57 (0.31-1.05, p = 0.073)
**Sperm source ${}^{\text{a}}$ **					
	**Self**	104 (72.7)	39 (27.3)	
	**Donor**	61 (73.5)	22 (26.5)	0.96 (0.52-1.77, p = 0.900)	0.94 (0.49-1.81, p = 0.857)
**Age ${}^{\text{b}}$ (yr)**		39.3 ± 6.2	37.8 ± 6.7	0.97 (0.92-1.01, p = 0.130)	1.00 (0.89-1.11, p = 0.929)
**BMI ${}^{\text{b}}$ **		26.1 ± 4.7	25.1 ± 3.1	0.94 (0.87-1.02, p = 0.145)	0.96 (0.88-1.04, p = 0.318)
**Indication ${}^{\text{a}}$ (RIF)**		21 (84.0)	4 (16.0)	0.71 (0.22-2.28, p = 0.565)	0.78 (0.21-2.89, p = 0.712)
**Endometrial thickness ${}^{\text{b}}$ **		9.6 ± 8.9	8.8 ± 1.2	0.98 (0.91-1.05, p = 0.541)	1.00 (0.94–1.07, p = 0.905)
${}^{\text{a}}$ Data shown as n (%), ${}^{\text{b}}$ Data shown as Mean ± SD. MODEL FIT: χ² (9) = 13.99, p = 0.123, Pseudo-R² = 0.05, Number in data frame = 226, Number in model = 226, Missing = 0, AIC = 269.6, C-statistic = 0.654, H&L = Chi-sq. (8) 8.93 (p = 0.348).OR: Odds ratio, CI: Confidence interval. Data presented as OR (95% CI). BMI: Body mass index, RIF: Recurrent implantation failure, Linear regression test					

**Table 5:** Association between donor groups and all parameters


**All parameters**	**Group**		**P-value**
	**1 (n = 109)**	**2 (n = 141)**	
**Age (yr)**	24.27 ± 2.17	24.74 ± 2.46	0.132
**BMI**	23.68 ± 1.99	23.41 ± 1.96	0.260
**Days of stimulation**	11.03 ± 0.80	11.00 ± 0.74	0.735
**Stimulation dose**	2428 ± 278.40	2251 ± 141.60	0.400
**Expected oocyte number**	17.03 ± 4.46	17.21 ± 5.26	0.943
**Retrieved oocyte number**	20.35 ± 10.89	18.79 ± 8.55	0.207
**Number of M-2 oocytes**	14.60 ± 8.26	13.40 ± 6.53	0.227
**Number of M-1 oocytes**	3.47 ± 1.95	3.26 ± 1.77	0.217
**Number of GV oocytes**	2.34 ± 1.77	2.15 ± 1.82	0.128
**Number of day-5 blastocysts formed**	5.96 ± 4.21	5.95 ± 3.72	0.835
**Number of good-quality blastocysts**	4.49 ± 3.28	4.21 ± 2.91	0.650
**Blastulation rate (%)**	39.09 ± 24.33	42.18 ± 22.88	0.165
**M-2 oocyte proportion (%)**	70.37 ± 9.35	71.17 ± 10.37	0.756
**Good-quality blastocyst proportion (%)**	30.61 ± 20.41	30.98 ± 18.90	0.779
Data are presented as Mean ± SD, Significant at p < 0.05. Wilcoxon Mann-Whitney U test. BMI: Body mass index, GV: Germinal vesicle			

**Table 6:** Regression of embryological outcomes with all variables in the model


**Dependent**		Value*	Coefficient**	
		**Univariable**	**Multivariable**
**M2 oocyte proportion percent**				
	**Group 1**	70.4 ± 9.3	- -
	**Group 2**	71.2 ± 10.4	0.79 (-1.74 to 3.32, p = 0.538)	-
	**Age**	70.8 ± 9.9	-0.24 (-0.78 to 0.30, p = 0.378)	-
	**BMI**	70.8 ± 9.9	0.32 (-0.32 to 0.96, p = 0.327)	-
	**Stimulation dose**	70.8 ± 9.9	-0.00 (-0.00 to 0.00, p = 0.180)	-
	**Retrieved oocyte number**	70.8 ± 9.9	0.17 (0.03 to 0.30, p = 0.016)	0.17 (0.03 to 0.30, p = 0.016)
**Good quality embryo proportion percent**				
	**Group 1**	30.6 ± 20.4	- -
	**Group 2**	31.0 ± 18.9	0.37 (-4.62 to 5.36, p = 0.883)	-
	**Age**	30.8 ± 19.5	0.95 (-0.11 to 2.00, p = 0.078)	-
	**BMI**	30.8 ± 19.5	-1.03 (-2.28 to 0.23, p = 0.108)	-0.99 (-2.22 to 0.24, p = 0.115)
	**Stimulation dose**	30.8 ± 19.5	0.01 (0.00 to 0.01, p = 0.002)	0.01 (0.00 to 0.01, p = 0.002)
	**Number of M2 oocytes**	30.8 ± 19.5	0.09 (-0.27 to 0.44, p = 0.631)	-
*Data presented as Mean ± SD. **Linear regression test, BMI: Body mass index, M2: Metaphase II				

## 4. Discussion

The use of GnRH antagonist vs. MPA had a comparable live birth in both groups. In this study, the authors found embryological characteristics to be comparable in both groups. Although live birth was comparable, with 2 embryos transferred in both the groups, twin sac implantations were significantly less in group 2. Comparative studies of progestin for prevention of LH surge, and its subsequent embryological and pregnancy outcomes in OS of patients followed by FET in the next cycle have shown similar results and comparable outcomes (2, 8). In a previous report of Beguería and colleagues, the duration of stimulation, as well as the total gonadotropin dose up to trigger, was similar in GnRH antagonist and MPA-treated patients (9).

As per the definition of premature LH surge, no premature LH surge was observed in other studies with the use of progestin to inhibit spontaneous ovulation during OS (10) or with the use of GnRH antagonists (11). In our study, MPA was started from day 2 of the menses along with rFSH injection. As a result, there was consistent LH suppression with no incidence of premature LH surge. In previous studies, there was no case of premature LH surge reported in PCOS patients receiving 10 mg of MPA daily as well as in normal responders co-administered with either 4 mg or 10 mg of MPA per day during OS (12, 13). We observed that embryological characteristics were similar in both groups. The M2 oocyte proportion showed slightly higher retrieval in group 2 but this was not statistically significant when multivariable regression analysis was performed, unlike the finding of significantly higher yield of M2 oocyte as stated in a retrospective study of donor oocyte cycles (14). It was a small study on 13 oocyte donors. Our finding is in accordance with the earlier comparative study where MPA was comparable to GnRH antagonist in terms of the number of mature oocytes retrieved at ovum pick up in oocyte donation cycles and was non-inferior to GnRH antagonist. This study comparing both drugs in the oocyte-donation cycle had comparable M2 retrieval but showed negative pregnancy outcomes in recipients (9); however, the intention-to-treat multilevel analyses had a p-value = 0.05 for live birth rate. In our study, live births were not significantly different in the groups. The effectiveness of MPA on the clinical pregnancy rates, implantation rates, and live-birth rates was previously studied and was found to be comparable (2, 13).

For proper interpretations of the results of pregnancy outcomes, one should keep in mind that, in most studies, a comparison of MPA was done with GnRH agonist or other progestins in short GnRH agonist protocols, rather than a GnRH antagonist protocol (2, 13). The current study, however, analyzed the results of the use of MPA in a GnRH antagonist protocol. The use of this protocol is recommended in oocyte donation cycles to reduce the incidence of ovarian hyperstimulation syndrome (15, 16). In addition, we administered rFSH to all patients and donors; in previous studies (15, 9), a few authors used hMG, having a small amount of urinary human chorionic gonadotropin (HCG) (13, 2). Moreover, we used the GnRH agonist trigger for donors to minimize the risk of ovarian hyperstimulation syndrome, as used in some studies (17, 18), while in other studies the trigger was performed with HCG (2) or by HCG and triptorelin (14).

Although vitrification of embryos is mandatory when used in patients, thereby increasing the cost of treatment, its cost-effectiveness, flexibility, and convenience cannot be denied in the oocyte donation cycle where FET is the protocol of choice. In India, most of the estimated 1.2 billion people have to pay healthcare expenses out of their own pocket. Less than 15% of the population has any kind of healthcare coverage, especially in rural areas. With a per capita income of $153 (in 2019-2020), any possible cost-effective alternative which is as efficient and comparable as conventional treatment protocols should be further explored. In this study, we have analyzed both self-sperm and donor-sperm as sperm sources that could have an impact on pregnancy outcomes. The difference in indication for IVF also could have contributed to pregnancy outcomes.

Previous studies have heterogeneity in the protocols used, so interpretations of results cannot be properly validated. Limited studies have compared GnRH antagonist vs. MPA in oocyte donation cycles. Further comparative studies that take into account all confounding factors with the use of a uniform and most feasible protocol, preferably prospective randomized controlled trials on larger populations, are required to validate the pregnancy outcomes with the use of MPA in OS protocols as opposed to those of conventional protocols during ART procedures, especially with oocyte donation cycles.

## 5. Conclusion

Based on the findings of this study, the authors conclude that MPA may be as effective as GnRH antagonists to achieve positive reproductive and pregnancy outcomes in the oocyte donation cycle.

##  Acknowledgments

The current study is not funded by any government or private organization. All authors have unanimously given consent and approval for the study with no conflict of interest.

##  Conflict of Interest

The authors declare that there is no conflict of interest.
